# Sr_2_Pt_8−*x*_As: a layered incommensurately modulated metal with saturated resistivity

**DOI:** 10.1107/S2052252518007303

**Published:** 2018-06-08

**Authors:** Edoardo Martino, Alla Arakcheeva, Gabriel Autès, Andrea Pisoni, Maja D. Bachmann, Kimberly A. Modic, Toni Helm, Oleg V. Yazyev, Philip J. W. Moll, László Forró, Sergiy Katrych

**Affiliations:** a Laboratory of Physics of Complex Matter, École Polytechnique Fédérale de Lausanne (EPFL), Lausanne CH-1015, Switzerland; b Max-Planck-Institute for Chemical Physics of Solids, Dresden 01187, Germany; c National Centre for Computational Design and Discovery of Novel Materials MARVEL, École Polytechnique Fédérale de Lausanne (EPFL), Lausanne CH-1015, Switzerland

**Keywords:** platinum-based metallic compounds, incommensurately modulated structure, vacancies, Mooij correlation, composite materials, inorganic materials, density functional theory

## Abstract

The high-pressure synthesis and incommensurately modulated structure are reported for the new compound Sr_2_Pt_8−*x*_As, with *x* = 0.715 (5). The structure consists of Sr_2_Pt_3_As layers alternating with Pt-only corrugated grids. *Ab initio* calculations predict a metallic character with a dominant role of the Pt *d* electrons. The electrical resistivity (ρ) and Seebeck coefficient confirm the metallic character, but surprisingly, ρ showed a near-flat temperature dependence. This observation fits the description of the Mooij correlation for electrical resistivity in disordered metals, originally developed for statistically distributed point defects. The discussed material has a long-range crystallographic order, but the high concentration of Pt vacancies, incommensurately ordered, strongly influences the electronic conduction properties. This result extends the range of validity of the Mooij correlation to long-range ordered incommensurately modulated vacancies. Motivated by the layered structure, the resistivity anisotropy was measured in a focused-ion-beam micro-fabricated well oriented single crystal. A low resistivity anisotropy indicates that the layers are electrically coupled and conduction channels along different directions are intermixed.

## Abstract 2

This article reports an investigation of the new compound Sr_2_Pt_8−*x*_As (*x* = 0.715), characterized by an incommensurately modulated structure and a temperature-independent electrical resistivity. The observation of saturated resistivity in this metallic system is described in the context of the Mooij correlation, which originates from the aperiodic potential of the incommensurately modulated vacancies in one of the Pt positions.

## 1. Introduction   

In the search for materials with novel electronic properties, the family of Pt-based ternary compounds is a good target because it is rich in various compositions, yet investigated only to a limited extent. In some, superconductivity and/or unconventional electronic properties have been reported, for example in SrPtAs (Nishikubo *et al.*, 2011 ▸), SrPt_2_As_2_ (Kudo *et al.*, 2010 ▸), SrPt_3_P (Takayama *et al.*, 2012 ▸), LaPt_5_As (Fujioka *et al.*, 2016 ▸), SrPt_6_P_2_ (Lv *et al.*, 2014 ▸), SrPt_2_Ge_2_ (Ku *et al.*, 2013 ▸), SrPtGe_3_ (Miliyanchuk *et al.*, 2011 ▸) and Ca_2_Pt_3_Si_5_ (Takeuchi *et al.*, 2009 ▸).

Our goal was to investigate the Sr–Pt–As ternary system in the Pt-rich area, which is largely unexplored. We started by targeting the hypothetical composition SrPt_3_As, not yet synthesized, but analogous to SrPt_3_P, which is known as a strongly coupled superconductor with a critical temperature *T*_C_ = 8.4 K (Takayama *et al.*, 2012 ▸) and characterized by a high electron–phonon coupling (Zocco *et al.*, 2015 ▸; Subedi *et al.*, 2013 ▸).

The synthesis of this composition, where As replaces P, was the primary motivation for this work. A similar strategy led to the discovery of high-temperature superconductivity (with a *T*_C_ up to 55 K) in the Fe-based pnictides, when LaO_1−*x*_F*_x_*FeAs was synthesized because of the known parent superconducting compound LaOFeP, which has a much lower critical temperature (*T*_C_ = 4 K) (Kamihara *et al.*, 2008 ▸).

We adopted a high-pressure synthesis route, through which we reproducibly synthesized the new compound Sr_2_Pt_8−*x*_As. It has a layered structure built from covalently bonded polyanions analogous to Ce_2_Pt_8_As (Chizhov *et al.*, 2009 ▸). The large number of ordered vacancies in Pt positions [*x* = 0.715 (5)] results in an incommensurately modulated (IM) structure. It is a fundamentally exciting question as to how these long-range ordered IM vacancies affect electronic transport properties. Do they act as random static defects which are at the origin of the Mooij correlation (Mooij, 1973 ▸)? Does the IM structure introduce a gap in the electronic states like in quasi-two-dimensional (Sipos *et al.*, 2008 ▸) or quasi-one-dimensional materials (Voit *et al.*, 2000 ▸)? To answer these questions, we performed a detailed refinement of the structure and calculated the density of states (DOS) using a density functional theory (DFT) approach. We also performed measurements of the electrical resistivity (ρ) and Seebeck coefficient (*S*) as a function of temperature. We did not observe the SrPt_3_As phase as a result of our high-pressure synthesis, despite fine variation in the parameter space of pressure, temperature and reaction time.

## 2. Experimental   

### 2.1. High-pressure synthesis   

The crystals were grown using a high-pressure multi-anvil setup because of the high arsenic partial pressure at high temperatures. Two preparation routes were used: (i) a pre-reacted mixture of a near-binary eutectic composition of Pt_7_As_3_ was combined with strontium and platinum, or (ii) the combination of pure elements: Pt sponge (60 mesh, 99.98%, metallic basis), Sr dendritic pieces (99.95%, metallic basis), powdered As sponge (99.95%, metallic basis). Details of each step are reported in Table 1 ▸. The mixture of Pt_7_As_3_ (2 g, prepared according to the procedure described in Table 1 ▸, No. 1) was compressed into a pellet with the addition of Pt and Sr (final composition: SrPt_3_As), sealed in a quartz glass tube at 202 hPa of Ar and heated according to No. 1*a*. The product was reground, compressed into a pellet and heated in a sealed quartz glass ampulla according to No. 1*b*. The product of No. 1*b* was placed in a boron nitride crucible and the crystals were grown by the self-flux method at high pressure (2.3 GPa, No. 1*c*). At the end of the synthesis, cooling to room temperature was realized by switching off the power supply.

### 2.2. X-ray diffraction   

**Figure 1:**
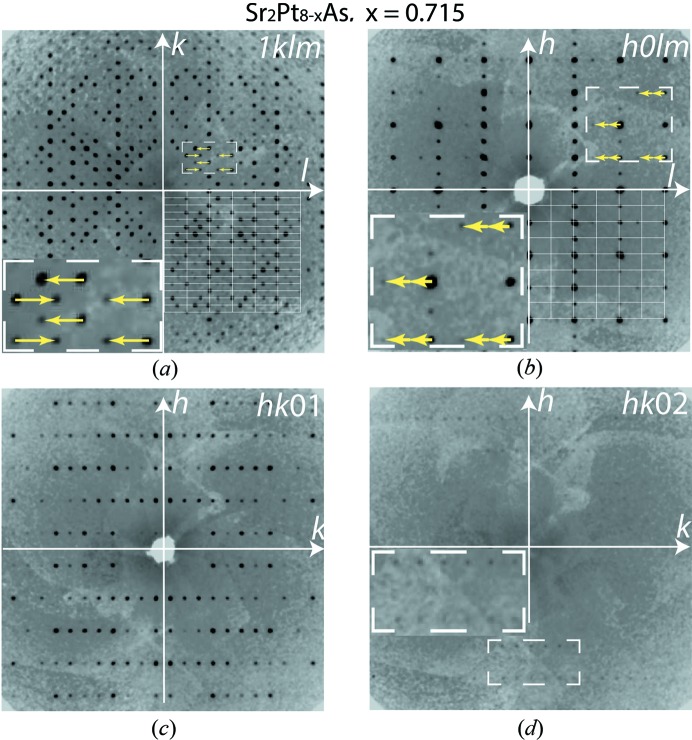
Sections of reciprocal space with main and satellite reflections. The *hklm* indices correspond to the orthorhombic unit-cell parameters *a* = 7.95, *b* = 18.10, *c* = 5.70 Å and the modulation wavevector **q** = 0.6038c* (yellow arrows in insets). In (*a*) and (*b*), the intersections of white lines in the bottom right-hand quarters define strong main *hkl*0 reflections; strong satellites of the first order, 1*kl*1, and weak satellites of the second order, *h*0*l*2, are away from the intersections. In (*c*) and (*d*), the *hk*01 and *hk*02 satellites are shown separately. Weak satellites of the second order, *hk*02, can be observed only in the areas with low background.

Single-crystal X-ray diffraction data collection was performed on a SuperNova (dual source) four-circle diffractometer (Agilent Technologies, USA) equipped with a CCD detector. Data reduction and analytical absorption correction were made using the *CrysAlisPRO* software package (Oxford Diffraction, 2014 ▸). The reciprocal space reconstructions supported the choice of the superspace group shown in Fig. 1 ▸. The crystal structure was solved by the charge-flipping method (Palatinus & Chapuis, 2007 ▸) and refined using the *JANA2006* program package (Petříček *et al.*, 2014 ▸). The outcome of the data analysis is given in Table 2 ▸, and further details are provided in the supporting information.

### 2.3. DOS calculations   

The first-principles calculation of the DOS was performed using DFT within the generalized gradient approximation as implemented in the *Quantum Espresso* package (Giannozzi *et al.*, 2009 ▸). The computation was carried out on the commensurate superstructure approximation of the solved structure with 204 atoms in the unit cell using scalar relativistic ultrasoft pseudo potentials, a 3 × 2 × 2 *k*-points mesh and a planewave kinetic energy cut-off of 50 Ry for the wavefunctions.

The projected DOS was obtained by projecting the Kohn–Sham wavefunctions onto localized atomic orbitals.

### 2.4. Electrical resistivity and Seebeck coefficient   

For precise measurements of the absolute electrical resistivity (ρ) and its anisotropy, we produced a micro-fabricated sample from a single crystal using a focused ion beam (FIB) (Moll, 2018 ▸). This approach has already proven its potential and reliability in the study of novel materials (Moll *et al.*, 2010 ▸). The starting crystal, which was 150 × 150 × 50 µm in size, was first analysed by single-crystal X-ray diffraction to confirm the correct structure and identify the crystallographic axes.

The Seebeck coefficient (*S*) is the magnitude of the open circuit voltage induced across a material under a thermal gradient, and was measured according to the previously reported procedure (Jaćimović *et al.*, 2013 ▸). For this experiment, we used a ceramic sample since *S* is not affected by grain boundaries, and the longer sample size guaranteed a well defined temperature gradient necessary for precise measurements. Both coefficients, ρ and *S* were measured in the 4.2–300 K temperature range.

## 3. Results   

### 3.1. Determination of the incommensurately modulated structure of Sr_2_Pt_8−*x*_As   

The distribution of reflections in reciprocal space is shown in Fig. 1 ▸, and in more detail in Fig. S1 (see supporting information). The main Bragg peaks correspond to orthorhombic symmetry, and the satellite reflections indicate an IM structure. The unit-cell parameters are *a* = 7.95, *b* = 18.10 and *c* = 5.70 Å, with the modulation wavevector **q** = 0.6038 (7)c*. The reflection conditions (*hklm*: *k* + *l* = 2*n*; 0*klm*: *m* = 2*n*; *h*0*lm*: *m* = 2*n*; *hk*00: *h* = 2*n*) point to the (3 + 1)-dimensional superspace group *Amma*(00γ)*ss*0 [No. 63.1.13.11 after van Smaalen *et al.* (2013 ▸) and Stokes *et al.* (2011 ▸)]. Two orders of the satellite reflections can be observed (Figs. 1 ▸ and S1), but second-order satellites are very weak, so the intensity (*I*) for only a few could be measured with meaningful significance [*I* > 3σ(*I*)]. Hence, only the first-order satellites were used in our calculations.

Based on main reflections only, the average structure was determined in the *Amma* space group (No. 63) with the reliability index *R* = 0.031. Five Pt, one Sr and one As site define the structure (Table S1), with the Pt5 site being partially occupied by about 68%. The low value of the reliability index for the average structure is a result of the specific structural modulations, which only slightly relate to the displacive modulations. Pt1 and As1 do not exhibit modulation because of symmetry restrictions; for a number of other atoms, allowed harmonics are zero within one standard deviation (Table S1). It should be noted that the very weak modulation of Pt5 along the *a* axis was constrained to zero because of the correlation with U11.

**Figure 2:**
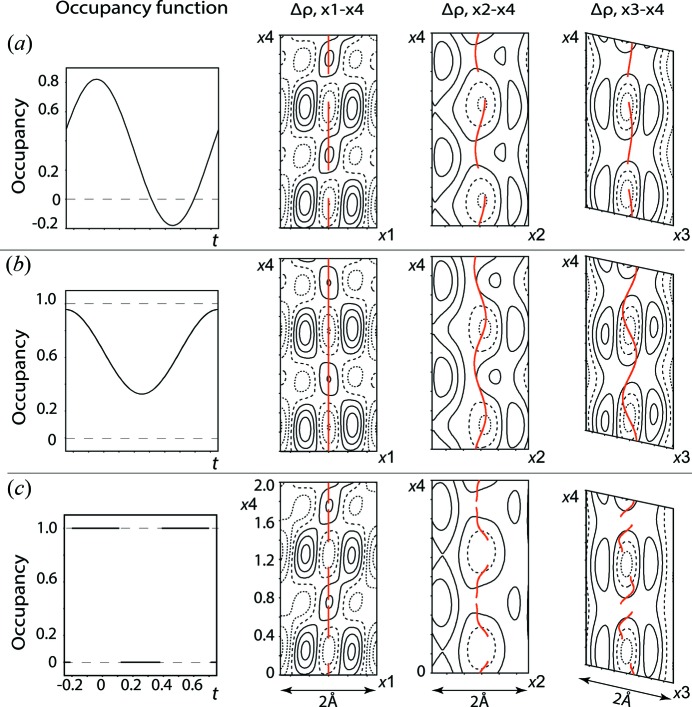
Three possible models of the Pt5 occupancy function and the corresponding Fourier maps of the residual electron density (Δρ) calculated for the vicinity of the Pt5 position. (*a*) The harmonic function applied without any restriction, (*b*) the harmonic function constrained by ocos1 = 0 and (*c*) the crenel function; all of them give low values of Δρ. Red lines show the position modulation functions of Pt5. The black solid, dashed and dotted lines indicate positive, zero and negative contours, respectively, with a step of 0.5 eÅ^−3^.

The strong intensities of the first-order satellite reflections (Fig. 1 ▸) are determined by the occupancy modulation of Pt5. Different possible models for the occupancy function are shown in Fig. 2 ▸. The harmonic function (Fig. 2 ▸ *a*) gives a negative occupancy of about 30% in the *t* range and a high uncertainty of the value for the cosine component of the occupancy wave, ocos1 = 0.48 (14). The negative occupancy indicates a high probability of the crenel model, which determines either the presence or absence of the atom along the *t* axis. The instability of the cosine component can be explained by the pseudo-special position of Pt5 (∼1/2, *y*, ∼0). The harmonic function gives satisfactory results only with the constraint ocos1 = 0 (Fig. 2 ▸ *b*). The crenel function (Fig. 2 ▸ *c*) gives reasonable results without any restriction. The residual electron density (Δρ) calculated in the vicinity of the Pt5 position is analogous for all the models (Fig. 2 ▸). All of them are characterized by the similar reliabilities (*R*_main_ ≃ 0.027, *R*_sat_ ≃ 0.038, *R*_all_ ≃ 0.030). A smaller Δρ_max_ (4 *versus* 4.6 e Å^−3^) and the absence of any restrictions favour the crenel model. Hence, we can conclude that this model is the more suitable approximation for the Pt5 occupancy function.

**Figure 3:**
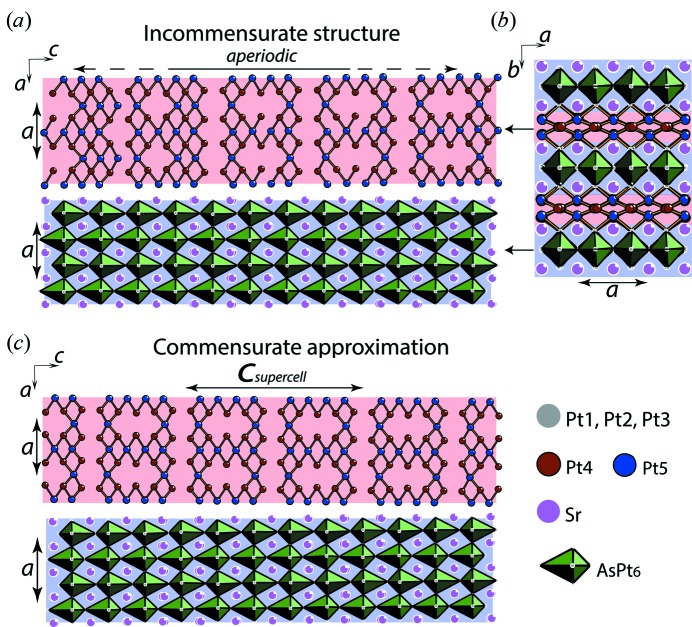
Sketch of the crystal structure of Sr_2_Pt_8−*x*_As. (*a*) and (*b*) are portions of the Sr_2_Pt_7.285_As IM structure with the modulation wavevector **q** = 0.6038c*. (*b*) and (*c*) represent the commensurate superstructure approximation with **q** = 0.6c*. Different colours show five different atomic sites of Pt, Pt1, Pt2 and Pt3 (grey) forming a layer of edge-sharing AsPt_6_ bipyramids (green) centred by As (blue background). This layer of AsPt_6_ bipyramids is identical in both the IM structure and its commensurate approximation. Pt4 (brown) and Pt5 (blue) form the ‘metallic’ corrugated grids (red background), which are aperiodic along the *c* axis in the IM structure. The short Pt–Pt contacts of 2.4–2.74 Å are shown by lines.

**Figure 4:**
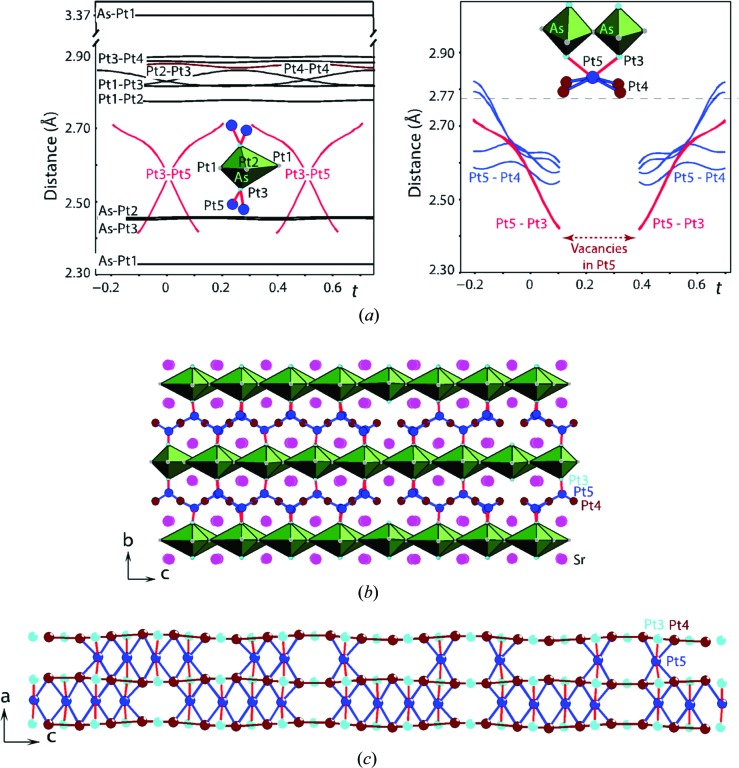
Interatomic distances in the Sr_2_Pt_7.285_As IM structure. (*a*) The *t* plot of the As–Pt and Pt–Pt distances. (*b*) A portion of the incommensurate structure with an indication of the most variable Pt5–Pt3 (red) and Pt5–Pt4 (blue) distances. (*c*) Variations of the Pt5–Pt3 and Pt5–Pt4 distances are determined by a wave of the Pt4 displacement along the *a* axis. The distances are indicated by identical colours in (*a*), (*b*) and (*c*).

Portions of the IM structure are shown in Figs. 3 ▸ and 4 ▸. Sr_2_Pt_8−*x*_As can be described as Sr_2_Pt_3_As layers (blue background in Fig. 3 ▸) alternating with the Pt-only corrugated grids (red background in Fig. 3 ▸). The atomic arrangement in the Sr_2_Pt_3_As layer is a host network of AsPt_6_ distorted bipyramids, with the As atom displaced along the apical direction (*c* axis in Fig. 3 ▸). The two apical distances are quite different, 2.33 and 3.37 Å (As–Pt1 in Fig. 4 ▸ *a*); however, the remaining four, corresponding to the bipyramid bases, are all 2.45 Å (two As–Pt3 and two As–Pt2 in Fig. 4 ▸).

Interatomic distances between neighbouring atoms show little variation in the IM structure, with the exception of those around Pt5 (Fig. 4 ▸ *b*). The Pt5–Pt(3,4) distances are not only the most variable, but they are also shorter than in pure Pt (2.77 Å). Moreover, as it can be deduced from Fig. 4 ▸(*b*) that vacancies in the Pt5 position occur at the minimum in the Pt5–Pt3 distance (<2.42 Å). In other words, the strain induced by shortening of the Pt5–Pt3 distances defines the vacancy positions (Janssen *et al.*, 2010 ▸). They are periodically located along the modulation wave (axis *t* in Fig. 4 ▸ *a*), but aperiodically, *i.e.* long-range ordered, in the bulk of the crystal.

### 3.2. Density of states   

**Figure 5:**
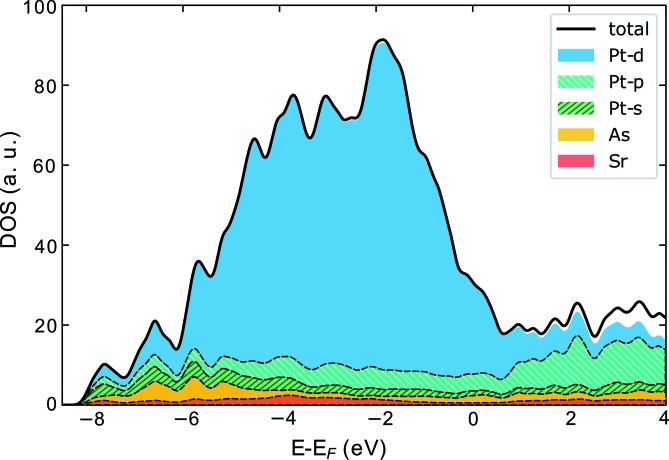
DOS calculated using DFT for the commensurate superstructure approximation, showing the contribution of each ion and Pt atomic orbital in a different colour. The high value of DOS at *E*_F_ indicates the metallic character of the compound.

In order to obtain insight into the electronic character of the compound, we calculated the DOS using DFT. The standard methodologies used to calculate electronic structures are not suitable for IM structures or structures with partially occupied sites. In order to provide a reasonable DOS estimation, we performed calculations on a commensurate superstructure approximation (Figs. 3 ▸ *b* and 3*c*). The experimental data were approximated using a modulation wavevector **q** = 3/5c* = 0.6c* (instead of 0.6038c*), which corresponds to the supercell parameter *c*_sc_ = 5*c* (Fig. 3 ▸ *c*). The two possible space groups, *Pnca* and *Pncn,* depending on the origin for the (3 + 1)-dimensional superspace (*t*_0_), were tested. The best results with the reliability index *R*(all) = 0.033 were obtained with the *Pncn* space group. Since the modulations in the displacement of all atoms are very small in the IM structure, the commensurate approximant differs only for the vacancy distribution within the Pt5 positions (Fig. 3 ▸ *c*). The composition of the approximant is Sr_2_Pt_7.2_As, slightly different from the real compound, Sr_2_Pt_7.285_As. For the commensurate analogue, the high DOS at the Fermi level (Fig. 5 ▸) clearly shows the metallic character of the compound and from the individual contribution of each ion, one can note that mainly the Pt *d* atomic orbitals contribute to electronic conduction, since they have the highest density at the Fermi level.

### 3.3. Anisotropy of the electrical resistivity and the Seebeck coefficient   

The manifestation of the high DOS seen above was investigated by means of electrical transport measurements. Initial measurements on ceramic samples from different syntheses showed a very weak temperature dependence of ρ (Fig. S2). In ceramic samples, crystallites are often randomly oriented and grain boundaries add an extrinsic contribution to the electrical resistivity, therefore we could not determine definitively whether the weak temperature dependence of ρ was an intrinsic property of the material.

**Figure 6:**
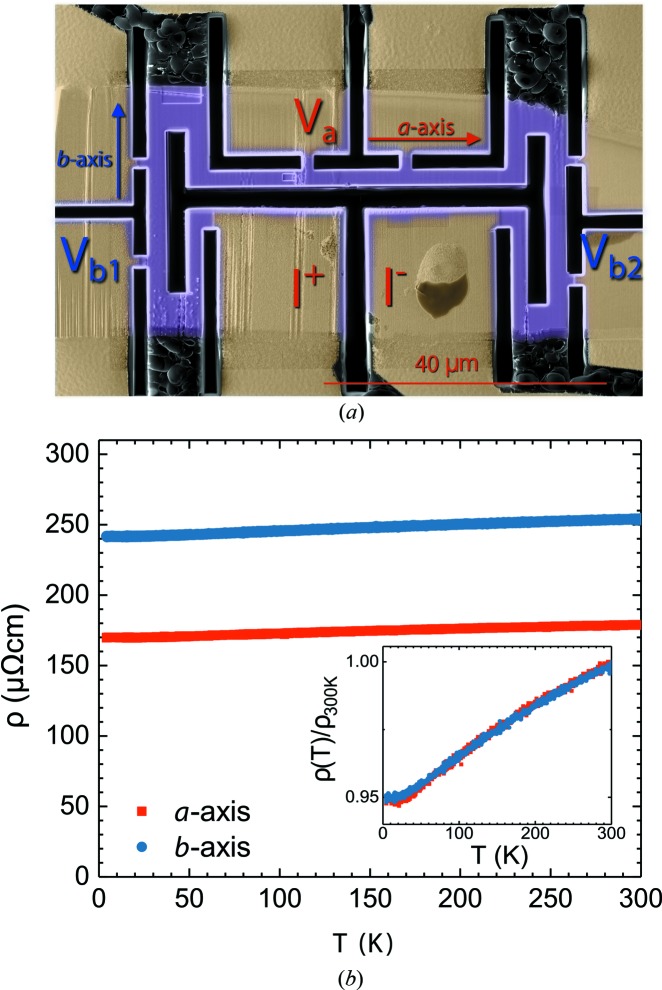
Electrical resistivity anisotropy measured on a micro-fabricated single crystal of Sr_2_Pt_8−*x*_As (purple in the SEM image). (*a*) The starting lamella were extracted from the single crystal following the identified crystallographic direction from X-ray diffraction. Electrical resistivity is measured along the *a* and *b* axes as a function of temperature. For the *b* axis, the voltage drop was measured at two positions with different geometrical factors (*V*_b1_, *V*_b2_). The current flows between the two leads (marked as I^+^ and I^−^). Colours are added to identify the micro-fabricated crystal (purple) and the sputtered gold top contacts (yellow). The crystal is fixed on a sapphire substrate by a drop of ep­oxy glue. (*b*) A nearly temperature-independent electrical resistivity was observed along both crystallographic directions. The resistivity is higher for current flow perpendicular to the layers. In the inset, resistivity curves normalized to their value at 300 K show identical temperature dependences.

The relevant measurements of ρ were obtained on a micro-fabricated and oriented single crystal, where the current paths along different crystallographic directions were designed with an FIB (see Fig. 6 ▸ *a*). The observation of weak temperature dependence, a 5% change from 300 to 4.2 K (Fig. 6 ▸ *b*), proves that this is an intrinsic property of the material, and not an artefact of grain boundary contribution or composition inhomogeneity. Thanks to the precise control of the sample geometry, the absolute value of 170 µΩ cm at 4.2 K for the resistivity along the *a* axis was determined with good accuracy.

These results (the large absolute value of ρ and its very weak temperature dependence) match very well the case of Mooij correlation, which shows a connection between the slope of the electrical resistivity (dρ/d*T*) and its absolute value in metals with a high a concentration of static defects (Mooij, 1973 ▸). Within the limit of low-defect concentration, the charge scattering on static defects adds a constant contribution to the electrical resistivity, described by Matthiessen’s rule, and simply shifts ρ(*T*) upwards. In the opposite case of high-defect concentration, the resistivity temperature dependence decreases, and above a threshold value (ρ_TH_) of 150–200 µΩ cm, its derivative (dρ/d*T*) changes sign from metallic (> 0) to non-metallic (< 0). This empirical relation between the slope and the value of the resistivity is known as the Mooij correlation, and it is closely related to the Ioffe–Regel criterion (Gurvitch, 1981 ▸) for metallicity and localization phenomena in metals. Such arguments have been developed for typical metallic systems, with carrier densities in the order of 10^23^ cm^−3^.

Belitz and Schirmacher went beyond the phenomenology and gave a theoretical description of the temperature dependence of the electrical resistivity in strongly disordered metals (Belitz & Schirmacher, 1983 ▸). In their work, they proposed the following equation: 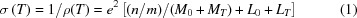
where *L*_0_ and *M*_0_ are the contributions to resistivity in the pristine sample, *M*_*T*_ is the generalized scattering rate attributed to the electron–phonon coupling and *L*_*T*_ is the phonon-assisted tunnelling rate of electrons. In their equation, the temperature dependence is given by electron–phonon scattering, as in any normal metal. The salient feature of strongly disordered systems is the occurrence of localizations, when the very short electron mean-free path localizes them at the same position. In this situation, scattering with phonons helps electrons to move, favouring the tunnelling between two distinct localization sites (*L*_*T*_). This mechanism is at the origin of ‘semiconducting-like’ resistivity in strongly disordered metals, since the phonon population, favouring electronic conduction, is thermally activated.

In the case of Sr_2_Pt_8−*x*_As, vacancies are long-range-ordered, with a modulation wavevector that is incommensurate with the crystal structure, a case so far not considered for the Mooij correlation. However, from the viewpoint of conduction electrons, vacancies ordered with an incommensurate periodicity act as scattering centres, like statistically distributed point defects. In our measurements, resistivity has a value close to ρ_TH_ along the plane, and even goes above that for the *b* axis, yet dρ/d*T* is positive, as in a metal (Fig. 6 ▸ *b*). However, there is no contradiction with the Mooij correlation, since Tsuei has shown that ρ_TH_ is not universal, the value at which the resistivity changes slope has a material specificity (Tsuei, 1986 ▸). This could very well apply to our compound.

A relevant analogy with our results can be drawn with the case of SrPt_3−*y*_Pd*_y_*P (Hu *et al.*, 2016 ▸). As pointed out in the crystal structure description, SrPt_3_P can be seen as a parent compound of Sr_2_Pt_8−*x*_As. Increasing Pd concentration in SrPt_3-*y*_Pd*_y_*P reduces the ρ temperature dependence to less than 5% from 300 to 6 K, resembling our experimental data for the case of higher substitution (*y* = 0.4), which is very close to the vacancy concentration for Pt5. We suppose that the same mechanism occurs in the two compounds, where defects in the Pt sites (vacancies or substitutions) strongly affect the electronic transport properties.

From the measurements on the micro-fabricated sample, electrical resistivity in the two perpendicular directions differs only in their absolute value (despite the layered structure), and both have the same temperature dependence, as can be seen in the inset in Fig. 6 ▸(*b*). One can say that from an electronic point of view, the material is isotropic. The scattering of the high-density Pt vacancies affects the electronic transport in the same way in the directions parallel and perpendicular to the layers, homogenizing the resistivity tensor to have identical temperature dependences along different directions.

**Figure 7:**
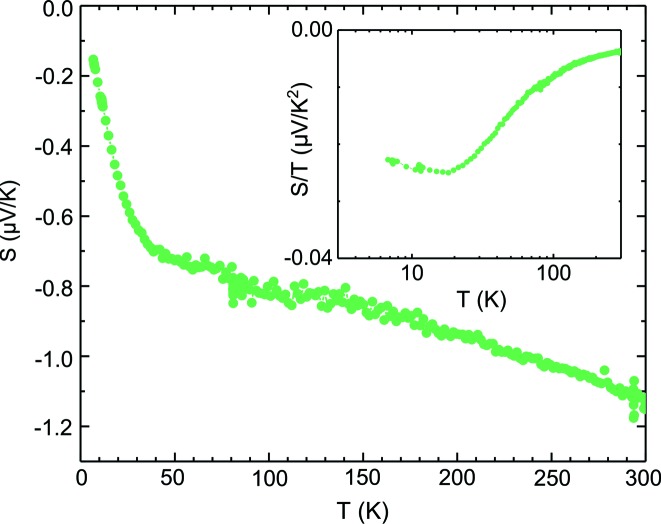
The temperature dependence of the Seebeck coefficient confirms the metallic character. The inset shows *S*/*T*, usually plotted for metallic systems.

The Seebeck coefficient measurement confirms experimentally the expected metallic character (Fig. 7 ▸), and the localization effects seen in resistivity are absent in *S*. The low absolute value and linear temperature dependence are the fingerprints of a large bandwidth metal (Behnia, 2015 ▸). *S* in a metallic system can be described by Mott’s formula: 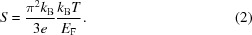
Following this equation, it is possible to approximate the Fermi energy from the linear part of *S*, evaluated to be in the 6 eV range. The inset of Fig. 7 shows *S*/*T*, which for metals is expected to be constant [see equation (2)]. Here it is temperature dependent, but this apparent non-metallicity is a result of the large intercept, probably coming from thermally activated additive contribution to *S* that freezes at temperatures lower than 50 K. The Seebeck coefficient at high temperatures (>50 K) can be described by the following equation: 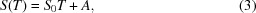
where *A* is the thermally activated temperature-independent additive contribution. Both *S*_0_ and *A* are negative.

## 4. Conclusions   

We have presented a new layered IM structure of Sr_2_Pt_8−*x*_As [*x* = 0.715(5)], synthesized at high pressure. High-quality X-ray diffraction data helped us to refine the structure of the crystal. The compound has a strong off-stoichiometry, and the structural strain field, acting on the aperiodically distributed vacancies, results in an IM structure. The incommensurate potential of long-range ordered Pt vacancies are responsible for the almost temperature-independent electrical resistivity, described in the context of the Mooij correlation. This experimental result shows that such correlation, first thought to occur only in the presence of stochastic disorder, can manifest also for long-range ordered IM vacancies, opening up new possibilities for theoretical studies and understanding of the phenomena of electron localization in metals.

A detailed understanding of the role of structure modulation in complex materials is an important issue and we believe that one can get a better insight by studying structurally well defined (Zhou *et al.*, 2016 ▸) crystalline materials with modulated structures similar to the present compound. For example, an important aspect of the physical properties would be the study of the optical conductivity. One could get an estimate of electronic relaxation time, or more importantly, if there is a resonance, a so-called pinned mode at finite energy that could manifest as a result of the incommensurate modulation.

Moreover, the analogous compound Ce_2_Pt_8_P (Chizhov *et al.*, 2009 ▸) belongs to a homological series of Ce*_n+m_*Pt_5*n+*3*m*_P*_m_* compounds, and because of its strong similarity to the investigated compound, the existence of the Sr*_n+m_*Pt_5*n+*3*m*_As*_m_* homological series is highly probable, suggesting that many more interesting compounds are still yet to be discovered.

## Supplementary Material

Crystal structure: contains datablock(s) global, I, II, III. DOI: 10.1107/S2052252518007303/fc5025sup1.cif

Supporting information file. DOI: 10.1107/S2052252518007303/fc5025sup2.pdf

CCDC references: 1843351, 1843352, 1843353

## Figures and Tables

**Table 1:** Details of the high-pressure synthesis of Sr_2_Pt_8−*x*_As single crystals

No.	Composition	Components	Procedure
1	Pt_7_As_3_	7Pt + 3As	0.5 h→673 K (1h†)→3 h→803 K (2 h)→1 h→853 K (5 h)→3 h→893 K (50 h)→WQ‡
1*a*	SrPt_3_As	⅓Pt_7_As_3_ (from No. 1) + ⅔Pt + Sr	4 h→873 K (1 h)→2 h→913 K (2 h)→1 h→923 K (2 h)→1 h→933 K (1 h)→1 h→943 K (2 h)→WQ
1*b*	SrPt_3_As	Product 1*a* powdered and compressed into a pellet	1 h→943 K (20 min)→2 h→1023 K (2 h)→1 h→1073 K (0.5 h)→2 h→1113 K (10 h)→WQ
1*c*	SrPt_3_As	Product 1*b* powdered and compressed into a pellet	2.3 GPa, 0.2 h →773 K→2 h→1423 K (0.7 h)→45 h→1323 K (20 h)→room temperature

† The annealing time in hours is given in parentheses.

‡ WQ, water quenched.

**Table 2:** Results of the X-ray study of Sr_2_Pt_8−*x*_As [*x* = 0.715 (5)]

Crystal data	
Chemical formula	AsPt_7.285_Sr_2_
*M*_r_	1671.32
Crystal system, superspace group	Orthorhombic, *Amma*(00γ)*ss*0† (No. 63.1.13.11‡)
Temperature (K)	293
Wavevectors	**q** = 0.6038 (7)c*
*a*, *b*, *c* (Å)	7.9509 (4), 18.1042 (10), 5.6972 (3)
*V* (Å^3^)	820.08 (8)
*Z*	4
Radiation type	Mo *K*α
μ (mm^−1^)	140.54
Crystal size (mm)	0.01 × 0.006 × 0.002

Data collection	
Diffractometer	SuperNova, Dual, Cu at zero, Atlas
Absorption correction	Multi-scan *CrysAlis PRO*, Agilent Technologies, Version 1.171.37.34. Empirical absorption correction using spherical harmonics, implemented in *SCALE3* *ABSPACK* scaling algorithm.
No. of measured, independent and observed [*I* > 3σ(*I*)] reflections	10459, 1220, 781
No. of observed reflections: main, the first-order satellites	440, 341
*R*_int_	0.092
(sin θ/λ)_max_ (Å^−1^)	0.625

Refinement	
*R*[*F* ^2^ > 3σ(*F* ^2^)], *wR*(*F* ^2^), *S*	0.0296, 0.0394, 1.32
*R, wR* for main reflections	0.0270, 0.0330
*R, wR* for satellites	0.0378, 0.0536
No. of reflections	1220
No. of parameters	54
Δρ_max_, Δρ_min_ (e Å^−3^)	3.99, −3.31

† No. of the superspace group in the superspace group table created by Stokes *et al.* (2011 ▸).

‡ Symmetry operations: (1) *x*_1_, *x*_2_, *x*_3_, *x*_4_; (2) −*x*_1_ + 1/2, −*x*_2_ + 1/2, *x*_3_ + 1/2, *x*_4_; (3) −*x*_1_, *x*_2_, −*x*_3_, −*x*_4_ + 1/2; (4) *x*_1_ + 1/2, −*x*_2_ + 1/2, −*x*_3_ + 1/2, −*x*_4_ + 1/2; (5) −*x*_1_, −*x*_2_ + 1/2, −*x*_3_ + 1/2, −*x*_4_; (6) *x*_1_ + 1/2, *x*_2_, −*x*_3_, −*x*_4_; (7) *x*_1_, −*x*_2_ + 1/2, *x*_3_ + 1/2, *x*_4_ + 1/2; (8) −*x*_1_ + 1/2, *x*_2_, *x*_3_, *x*_4_ + 1/2.
