# Supplementary material for: Sr2Pt8−xAs: a layered incommensurately modulated metal with saturated resistivity
Source: IUCrJ. 2018 Jun 8;5(Pt 4):470–7. doi: 10.1107/S2052252518007303 (PMC6038961; doi:10.1107/S2052252518007303)
Supplement: Supplementary file 2 [file m-05-00470-sup2.pdf]

IUCrJ

Volume 5 (2018)

Supporting information for article:

Sr2Pt8-*x*As: a layered incommensurately modulated metal with saturated resistivity

Edoardo Martino, Alla Arakcheeva, Gabriel Autès, Andrea Pisoni, Maja D. Bachmann, Kimberly A. Modic, Toni Helm, Oleg V. Yazyev, Philip J. W. Moll, László Forró and Sergiy Katrych

Table S1

Table S2 Atomic position parameters. The final coordinates, equivalent displacement parameters and Fourier amplitudes of the displacive modulation function for $Sr_2Pt_{7.285}As$. The waves are sorted by the term s for sines, c for cosines and order n.

Atom	Occupancy	Wave of modulation	*x*	*y*	*z*	U_{eqv}
Pt1	1.0		0.25	0.5	0.33957(13)	0.0043(2)
		s,1	0	0	0	
		c,1	0	0	0	
Pt2	1.0		0	0.5	0	0.0049(2)
		s,1	0	0.0081(6)	0	
		c,1	0	0	0	
Pt3	1.0		0.25	0.38865(3)	-0.00543(9)	0.0049(2)
		s,1	-0.01479(9)	0	0	
		c,1	0.0000(2)	0	0	
Pt4	1.0		0.25	0.25139(3)	0.25001(9)	0.0068(2)
		s,1	-0.0030(2)	0	0	
		c,1	-0.0278(1)	0	0	
Pt5†	0.642(2)		0.5000(2)	0.29822(5)	0.0128(5)	0.0052(5)
		s,1	0††	0.00564(11)	0.017(3)	
		c,1	0††	-0.0022(4)	0.0154(9)	
Sr1	1.0		0	0.87900(8)	0	0.0066(4)
		s,1	0	0.00158(9)	0	
		c,1	0.0000(4)	0	0.0284(3)	
As1	1.0		0.25	0.5	0.7485(3)	0.0024(5)
		s,1	0	0	0	
		c,1	0	0	0	

† The atom occupancy is represent by the crenel function with $x4_0$ = 0.9606(5) and $\Delta x4$ = 0.3212(13).

†† s,1 and c, 1 were constrained to be 0.

Table S3 The ADP harmonic parameters of atoms in $Sr_2Pt_{7.285}As$.

Atom	U11	U22	U33	U12	U13	U23
Pt1	0.0052(3)	0.0049(4)	0.0028(4)	0	0	0
Pt2	0.0007(4)	0.0089(4)	0.0051(4)	0	0.0000(2)	0
Pt3	0.0071(3)	0.0020(3)	0.0057(3)	0	0	0.00006(19)
Pt4	0.0121(3)	0.0041(3)	0.0044(3)	0	0	0.0017(2)
Pt5	0.0029(4)	0.0037(5)	0.0090(12)	0.0001(5)	0.0001(3)	-0.0023(9)
Sr1	0.0024(6)	0.0057(8)	0.0117(7)	0	-0.0006(5)	0
As1	0.0011(8)	0.0035(10)	0.0026(9)	0	0	0

Table S4 Selected interatomic distances (Å) for $Sr_2Pt_{7.285}As$.

Atom-Atom distance	Average	Minimal	Maximal
Pt1-Pt2	2.7757(10)	2.7738(10)	2.7776(10)
Pt1-Pt2(i)	2.7757(10)	2.7738(10)	2.7776(10)
Pt1-Pt3	2.8168(7)	2.8156(7)	2.8180(7)
Pt1-Pt3(i)	2.8168(7)	2.8156(7)	2.8180(7)
Pt2-Pt3	2.8343(13)	2.8157(14)	2.8588(14)
Pt2-Pt3(i)	2.8343(13)	2.8157(14)	2.8588(14)
Pt2-Pt3(ii)	2.8343(13)	2.8157(14)	2.8588(14)
Pt2-Pt3(iii)	2.8343(13)	2.8157(14)	2.8588(14)
Pt3-Pt4	2.8818(7)	2.8798(7)	2.8838(7)
Pt3-Pt4(vi)	2.8945(7)	2.8929(7)	2.8961(7)
Pt3-Pt5	2.599(4)	2.419(5)	2.713(5)
Pt3-Pt5(vii)	2.597(4)	2.416(5)	2.717(5)
Pt3-Pt5(viii)	2.596(4)	2.416(5)	2.717(5)
Pt3-Pt5(ix)	2.600(4)	2.419(5)	2.713(5)
Pt4-Pt4(vi)	2.8550(7)	2.8490(8)	2.8608(8)
Pt4-Pt4(x)	2.8549(7)	2.8491(8)	2.8608(8)
Pt4-Pt5	2.674(5)	2.597(8)	2.792(8)
Pt4-Pt5(x)	2.619(6)	2.571(7)	2.650(7)
Pt4-Pt5(vii)	2.576(6)	2.539(7)	2.601(7)

Pt4-Pt5(xi)	2.675(5)	2.585(8)	2.820(8)
Pt4-Pt5(xii)	2.675(5)	2.585(8)	2.820(8)
Pt4-Pt5(viii)	2.576(6)	2.539(7)	2.601(7)
Pt4-Pt5(xiii)	2.620(6)	2.571(7)	2.650(7)
Pt4-Pt5(ix)	2.675(5)	2.597(8)	2.792(8)
Pt5-Sr1(xiv)	3.213(8)	2.987(12)	3.309(12)
As1-Pt1	2.330(2)	2.330(2)	2.330(2)
As1-Pt1(xv)	3.368(2)	3.368(2)	3.368(2)
As1-Pt2(xv)	2.4526(15)	2.4505(15)	2.4548(15)
As1-Pt2(xvi)	2.4526(15)	2.4505(15)	2.4548(15)
As1-Pt3(xv)	2.4570(12)	2.4556(12)	2.4584(12)
As1-Pt3(xvi)	2.4570(12)	2.4556(12)	2.4584(12)

(i) -x+1/2,-y+2,z+1

(ii) -x,y,-z

(iii) x-1/2,-y+2,-z+1

(iv) x,y,z-1

(v) -x,y,-z+1

(vi) -x+1/2,-y+1/2,z-1/2

(vii) -x+1,y,-z

(viii) x-1/2,y,-z

(ix) -x+1/2,y,z

(x) -x+1/2,-y+1/2,z+1/2

(xi) x-1/2,-y+1/2,-z+1/2

(xii) -x+1,-y+1/2,-z+1/2

(xiii) x,-y+1/2,z+1/2

(xiv) x+1/2,y-1/2,-z-1/2

(xv) x, y, z+1

(xvi) -x+1/2, -y+2, z+2

1 Reflections in the reciprocal space

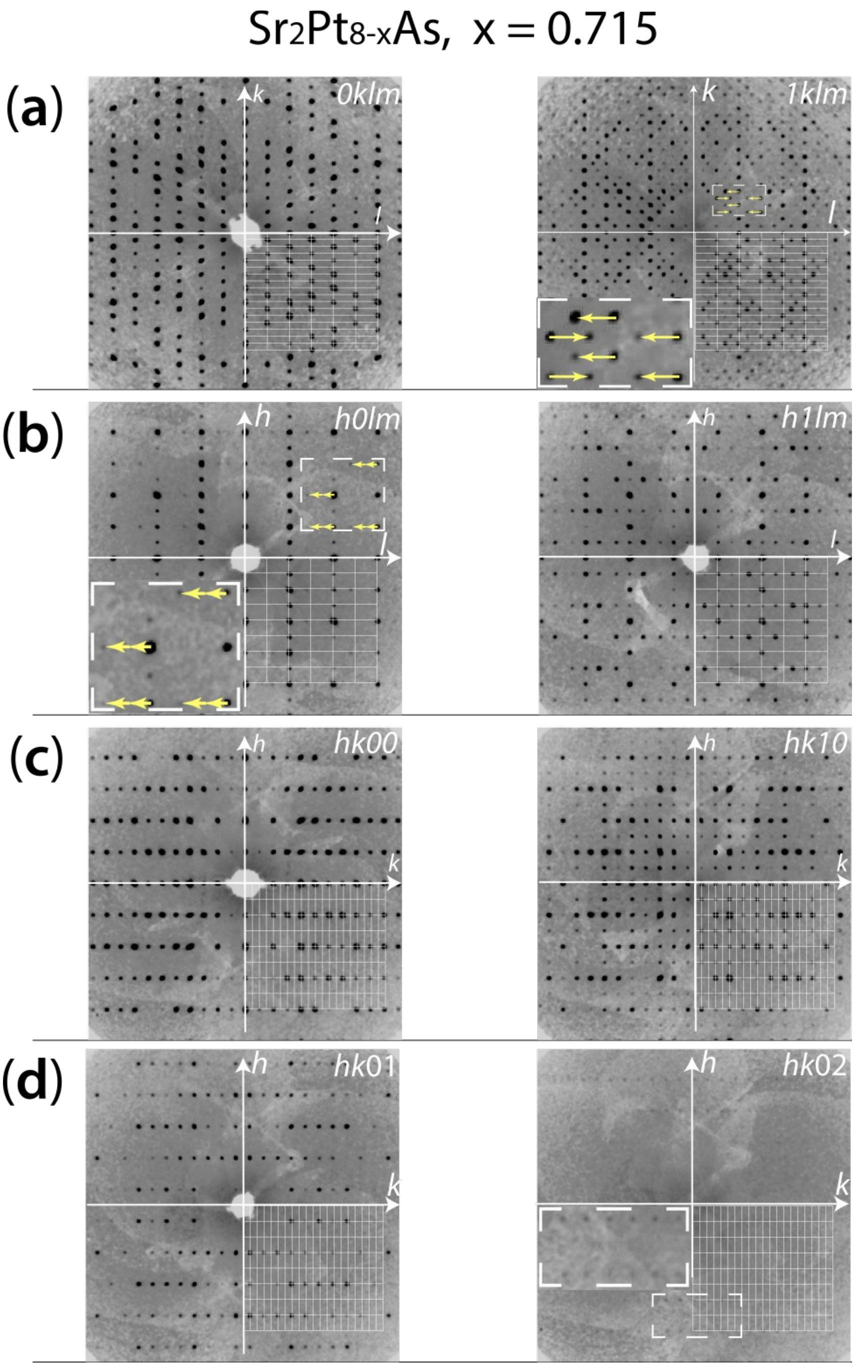

Figure S1 Sections of reciprocal space with indexation of reflections. The reflection indices correspond to the orthorhombic unit cell parameters **a** = 7.95, **b** = 18.10, **c** = 5.7 Å and the modulation wave vector **q** = 0.396**c*** (yellow arrows in inset). In section (a), (b), and (c): the intersections of grey lines in the right-top quarter define strong main hkl0 reflections; strong satellites of the 1st order, hkl1, and very weak satellites of the 2nd order, hkl2, are out of the intersections. (d) The hk01 and hk02 satellites are shown separately in the hk0.396 and hk0.792 sections, respectively.

2 Transport measurements on ceramic samples

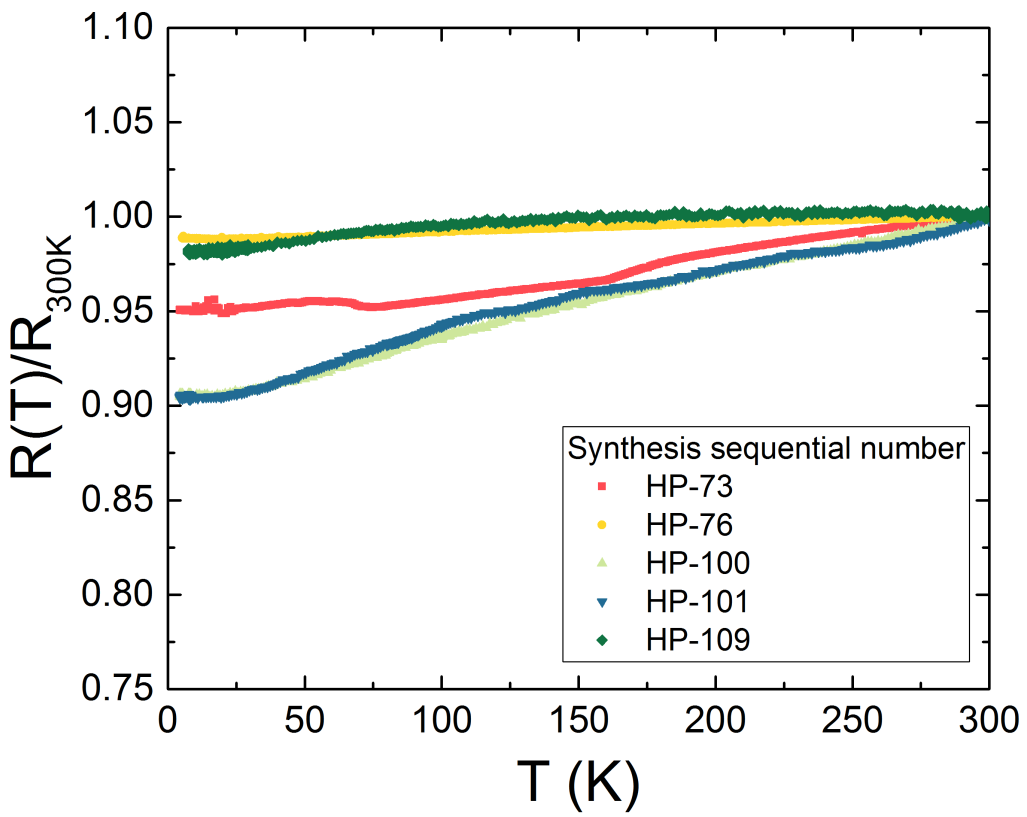

Figure S2 Electrical reactance normalized at room temperature (300 K), measured on fragments of the ceramic samples as received from high-pressure synthesis. The lack of control in the sample geometry and orientation hinders the precise evaluation of resistivity absolute value. Different batches (reported with a code name) show similar weak temperature dependence and positive dρ/dT.
